# Comparison of uterine preservation versus hysterectomy in women with placenta accreta: A cross-sectional study

**DOI:** 10.18502/ijrm.v20i9.12063

**Published:** 2022-10-10

**Authors:** Razieh Mohammad Jafari, Mahin Najafian, Mojgan Barati, Najmieh Saadati, Zorvan Jalili, Atefeh Poolad

**Affiliations:** ^1^Department of Obstetrics and Gynecology, School of Medicine, Fertility Infertility and Perinatology Research Center, Ahvaz Jundishapur University of Medical Sciences, Ahvaz, Iran.; ^2^Department of Obstetrics and Gynecology, School of Medicine, Ahvaz Jundishapur University of Medical Sciences, Ahvaz, Iran.

**Keywords:** Placenta accreta, Placenta diseases, Pregnancy complications, Conservative treatment, Hysterectomy.

## Abstract

**Background:**

Placenta accreta spectrum (PAS) is a major cause of obstetric bleeding in third trimester of pregnancy.

**Objective:**

This study aimed to compare the outcomes of uterine preservation surgery vs. hysterectomy in women with PAS.

**Materials and Methods:**

In this retrospective cross-sectional study, the records of 68 women with PAS referred to the Imam Khomeini hospital in Ahvaz, Iran, between March 2015 and February 2020 were included. The women were divided into 2 groups according to surgical approach: hysterectomy vs. uterine preservation (including just removing the lower segment, removing the lower segment with uterine artery ligation, or removing the lower segment with hypogastric artery ligation during cesarean section). The need for blood components transfusion (whole blood, packed cells, and fresh frozen plasma), maternal mortality, duration of surgery, and length of hospitalization were compared between groups.

**Results:**

In total, we investigated 68 women between the ages of 24-45 yr (mean age of 32.88 ± 5.08 yr). All participants were multiparous and underwent cesarean section. Furthermore, 28 women (41.2%) had a history of curettage. In total, 24 women (35.3%) underwent a hysterectomy, and 44 (64.7%) underwent uterine preservative surgeries. There were no significant differences between groups of hysterectomy and uterine preservative surgeries in terms of the need for blood components transfusion, maternal mortality, duration of surgery, and length of hospitalization.

**Conclusion:**

The results of this study showed no significant difference between groups regarding the studied outcomes. Therefore, conservative surgeries could be used to preserve the uterus instead of hysterectomy in women with PAS.

## 1. Introduction

Abnormal adhesion of the placenta involves the invasion of placental tissue into the myometrium and even serosis of the uterus, extending beyond the uterus and invading adjacent organs such as the bladder and intestines (1). The degree of placental invasion in the myometrium consists of 3 levels: the placenta accreta, increta, and percreta accrete, all known as the placenta accreta spectrum (PAS) (2). Placenta accreta has an overall incidence of approximately 1 in 500, and its occurrence is closely related to having had previous uterus surgeries (3). Furthermore, with the high prevalence of cesarean delivery, the incidence of placenta accreta has increased significantly in recent years (4).

The main issue with this type of pregnancy complication is massive bleeding during cesarean section (CS) and the necessity of hysterectomy and blood transfusion, which may exacerbate the patient's condition through complications such as bladder and ureter injuries, disseminated intravascular coagulation, massive blood transfusion, and even maternal death (5). Different therapeutic procedures can be used to control the bleeding in these participants, including hysterectomy and conservative approaches such as uterine and hypogastric artery ligation, methotrexate injections, and uterine artery ligation (6). Unfortunately, there has been no consensus on how to manage the massive bleeding problem.

The American College of Obstetricians and Gynecologists recommends CS hysterectomy as the primary treatment for PAS (7). However, hysterectomy can have significant complications, such as permanent loss of fertility and damage to the bladder and vascular structure (8). Maintaining fertility is essential for young pregnant women with PAS. Thus, hysterectomy cannot be the first-line approach because participants usually require a conservative approach that can preserve the uterus (9).

Because there is no cure for placenta accreta, contemporary management methods include intense procedures such as elective cesarean hysterectomy, compression sutures, myometrial excision, and leaving the placenta in situ (10, 11). Uterine preservation is particularly important in young reproductive-aged women. Uterine artery ligation is one of the methods that has been recently proposed to prevent or treat severe bleeding during CS and to try to preserve the uterus in these participants. Effective procedures must be adopted immediately to deal with this life-threatening condition (9).

Due to the various pregnancy complications of placenta accreta and the lack of consensus on the type of treatment, this study aimed to evaluate and compare the complications of conservative and hysterectomy treatments in PAS patients referred to the Imam Khomeini hospital in Ahvaz, Iran.

## 2. Materials and Methods

In this retrospective cross-sectional study, the records of 68 pregnant women with PAS referred to the Imam Khomeini hospital, Ahvaz, Iran, between March 2015 and February 2020 were studied in 2 groups: group I (CS + hysterectomy) and group II (uterus preservation approaches including removal of the lower segment only, or removal of the lower segment with uterine artery ligation, or removal of the lower segment with hypogastric artery ligation). Women with PAS shown on their obstetric ultrasound or with placental invasion at the time of surgery were included. Women with incomplete medical records were excluded from this study.

Confirmation of the diagnosis was based on the presence of placental tissue on the serous surface of the uterus or abnormal adhesion of the placenta after manual resection in the operation report. Data were extracted from the participants' medical records on their birth date, parity, gravidity, history of abortion, CS, curettage, myomectomy, ultrasound results, initial diagnosis, the need for blood components transfusion (whole blood, packed cells, and fresh frozen plasma [FFP]), maternal mortality, and the duration of surgery and hospitalization, and these data were compared between the 2 groups.

### Ethical considerations

This study was approved by the Ethical Committee of Ahvaz University of Medical Sciences and Health Services, Ahvaz, Iran (Code: IR.AJUMS.REC.1398.971). The identities of all participants remained confidential.

### Statistical analysis

The continuous variables were described using the mean ± standard deviation. For normality checks, the Kolmogorov-Smirnov and Shapiro-Wilk tests were used. To assess the normally distributed data, the student's *t* test was utilized. The Mann-Whitney U test was used for intergroup comparisons to evaluate the non-normally distributed data. The IBM Statistical Package for the Social Sciences (SPSS, version 22, SPSS Inc., Chicago, IL, USA) was used to perform all analyses. P-values < 0.05 were determined as significant.

## 3. Results

A total of 68 women with PAS were studied in 2 groups: group I (n = 24; 35.3%) and group II (n = 44; 64.7%). The mean age of participants was 32.88 ± 5.08 yr (range: 24-45). There were no significant differences between the 2 groups regarding age, gravidity, history of abortion, CS, curettage, and myomectomy (Table I). All participants were multiparous and had at least 2 pregnancy experiences with a range of 2-8 for gravidity. In this study, delivery was performed by CS in all participants. Also, 28 (41.2%) participants had a history of curettage.

The duration of hospitalization in most patients was between 1-5 days, and the duration of surgery in most patients was between 60, and 120 mins in both groups.

In group II, out of the 44 women who underwent preservative surgery, 7 women (10.3%) underwent CS and removal of the lower segment, 28 (41.2%) underwent CS and removal of the lower segment with uterine artery ligation, and 9 (13.2%) underwent CS and removal of the lower segment with hypogastric artery ligation. The study results did not show significant differences between the 2 groups regarding the need for blood components transfusion (whole blood, packed cells, and FFP), maternal mortality, or duration of surgery and hospitalization (Table II).

**Table 1:** Descriptive data of demographic characteristics, clinical manifestations, and complications


**Variables**	**Group I (n = 24)**	**Group II (n = 44)**	**P-value**
Age (yr)*	32.54 ± 5.69	33.23 ± 4.72	0.59
Gravidity*	3 ± 1	4 ± 2	0.60
Abortion**	6 (25.00)	14 (31.82)	0.55
History of curettage**	8 (33.33)	20 (45.45)	0.33
History of myomectomy**	0 (0)	0 (0)	1.00
History of cesarean section**	24 (100)	44 (100)	1.00
*Data presented as Mean ± SD. **Data presented as n (%). Independent sample *t* test. Group I: CS + hysterectomy, Group II: CS + Preservative surgery			

**Table 2:** Comparison of clinical outcomes between the 2 study groups


**Variables**		**Group I (n = 24)**	**Group II (n = 44)**	**P-value**
**Maternal mortality**		2 (8.33)	1 (2.27)	0.25
**Need for blood components transfusion**		17 (70.83)	33 (75.00)	0.57
**Fresh frozen plasma** **transfusion**		2 (8.33)	5 (11.36)	0.54
**Duration of hospitalization (days)**				
	**1-5 **	16 (66.66)	35 (79.54)	
	**6-10**	6 (25.20)	7 (15.92)	
	**11-15**	1 (4.16)	1 (2.27)	
	**16-20**	1 (4.16)	1 (2.27)	0.64
**Duration of surgery (min)**				
	**< 60 **	3 (12.50)	11 (25.00)		
	**60-119 **	15 (62.50)	25 (56.82)		
	**120-179**	5 (20.83)	7 (15.91)	
	**180-239**	1 (4.17)	1 (2.27)	
	**< 240**	0 (0)	0 (0)	0.73
Data presented as n (%). Mann-Whitney U test. Group I: CS + hysterectomy, Group II: CS + preservative surgery				

## 4. Discussion 

In this study, we aimed to compare the 2 operative treatments for placenta accreta and found no significant difference between the 2 groups in terms of the need for blood components transfusion (whole blood, packed cells, and FFP), maternal mortality, duration of surgery, and hospitalization stay.

Despite many advances in diagnosing placental abnormalities, PAS is still associated with high mortality and morbidity (12). Since maternal mortality is a crucial aspect of development according to the World Health Organization and other international institutions (7), our objective was to evaluate placenta accreta management strategies at the Imam Khomeini hospital in Ahvaz, Iran.

In this study, all participants were multiparous, and delivery was by CS in all individuals, consistent with previous studies (13-16). In a previous study that examined variables affecting placenta previa, a strong association was identified between a history of CS, a history of induced abortion, and a history of placenta previa (14). In our study, all participants had a history of CS. Despite other risk factors, CS is by far the most influential. The number of previous times that a pregnant woman has experienced CS is directly associated with the development of aberrant placenta, and the risk is higher in women who have had placenta accreta (16).

There are many differences in the definitions of PAS disorders used by specialists. One study showed that hysterectomy was the most common choice of treatment for PAS among the specialists studied (61%) (17). In another study, hysterectomy was the first treatment choice for PAS patients (18). In a systematic cohort study of high-risk women with PAS disorders, hysterectomy was performed in 208 cases, and conservative surgery was performed in 7 cases with local resection of the PAS myometrium (19). However, in our study, conservative surgery was the most common method used by the specialists (63.8%). Among the conservative surgeries, 28 cases (41.2%) underwent a CS and resection of the lower section by closing the uterine artery, the most common approach in this group.

In the present study, there was no significant difference between the 2 groups regarding the need for blood components transfusion (whole blood, packed cells, and FFP), which is consistent with previous study (20). Although blood products can save lives, they can also be associated with critical maternal complications and can lead to maternal death (21). Also, one of the essential indicators in determining maternal complications is the duration of surgery. Women who have longer surgeries need longer hospital stays (22). In one study the hysterectomy group had a higher morbidity rate than the conservative uterine surgery groups. There were also significant differences in operative time, the amount of transfused blood products including the number of red blood cells and FFP, and length of postoperative intensive care unit and hospital stays, which is inconsistent with our findings (23). These differences in the results can be due to differences in the patients characteristics, treatment modality, PAS severity and also the experience of the surgeons. Due to the need to maintain the reproductive capacity of individuals, several surgical methods have been introduced to preserve the uterus. Conservative therapies show fewer adverse effects than hysterectomies (8, 23–25). For example, one study found that when using the resective-reconstructive technique, in 80% of cases, bleeding was reduced in addition to preserving the uterus (25). However, due to the differences in degrees of PAS disorders and the existence of other conservative treatments, there is a need for further studies and reviews of these treatments in standardized conditions.

In general, alternative conservative therapies to preserve the uterus also increase the chance that the woman will be able to reproduce in the future, which affects a person's social status and self-esteem (26). However, compared to cesarean hysterectomy, the conservative approach's major drawback is the length of therapy and the necessity for long-term follow-up. Conservative management is a valid treatment option for patients and could be performed based on their clinical conditions.

## 5. Conclusion

This study showed no significant difference between the 2 groups regarding the need for blood components transfusion (whole blood, packed cells, and FFP), maternal mortality, and duration of surgery and hospitalization. Based on these results, conservative surgeries can be used instead of hysterectomy in patients with PAS, which can help to maintain fertility.

##  Conflict of interest

The authors declare that there is no conflict of interest.
